# Ellagic Acid as a Potential Inhibitor against the Nonstructural Protein NS3 Helicase of Zika Virus: A Molecular Modelling Study

**DOI:** 10.1155/2022/2044577

**Published:** 2022-08-21

**Authors:** Malathi Kullappan, Balakrishnan Anna Benedict, Anusha Rajajagadeesan, Padmasini Baskaran, Nanthini Devi Periadurai, Jenifer Mallavarpu Ambrose, Sri Harshini Gandhamaneni, Aruna Kumari Nakkella, Alok Agarwal, Vishnu Priya Veeraraghavan, Krishna Mohan Surapaneni

**Affiliations:** ^1^Department of Research, Panimalar Medical College Hospital & Research Institute, Varadharajapuram, Poonamallee, Chennai 600 123, India; ^2^Department of Chemistry, Panimalar Institute of Technology, Poonamallee, Chennai, 600 123 Tamil Nadu, India; ^3^Department of Biochemistry, Panimalar Medical College Hospital & Research Institute, Varadharajapuram, Poonamallee, Chennai 600 123, India; ^4^Department of Emergency Medicine, Panimalar Medical College Hospital & Research Institute, Varadharajapuram, Chennai, 600 123 Tamil Nadu, India; ^5^Departments of Microbiology and Molecular Virology, Panimalar Medical College Hospital & Research Institute, Varadharajapuram, Poonamallee, Chennai 600 123, India; ^6^Department of General Medicine, Panimalar Medical College Hospital & Research Institute, Varadharajapuram, Chennai, 600 123 Tamil Nadu, India; ^7^Department of Engineering Chemistry, Dr. B R Ambedkar University, Etcherla, Srikakulam, 532 410 Andhra Pradesh, India; ^8^Department of Chemistry, Chinmaya Degree College, BHEL, Haridwar, 249403 Uttarakhand, India; ^9^Department of Biochemistry, Saveetha Dental College, Saveetha Institute of Medical and Technical Sciences (SIMATS), Saveetha University, Chennai, Tamil Nadu, India; ^10^Departments of Biochemistry, Molecular Virology, Research, Clinical Skills & Simulation, Panimalar Medical College Hospital & Research Institute, Varadharajapuram, Poonamallee, Chennai, 600 123 Tamil Nadu, India

## Abstract

Zika virus is a member of the Flaviviridae family and genus Flavivirus, which has a phylogenetic relationship with spondweni virus. It spreads to humans through a mosquito bite. To identify potential inhibitors for the Zika virus with biosafety, we selected natural antiviral compounds isolated from plant sources and screened against NS3 helicase of the Zika virus. The enzymatic activity of the NS3 helicase is associated with the C-terminal region and is concerned with RNA synthesis and genome replication. It serves as a crucial target for the Zika virus. We carried out molecular docking for the target NS3 helicase against the selected 25 phytochemicals using AutoDock Vina software. Among the 25 plant compounds, we identified NS3 helicase-ellagic acid (-9.9 kcal/mol), NS3 helicase-hypericin (-9.8 kcal/mol), and NS3 helicase-pentagalloylglucose (-9.5 kcal/mol) as the best binding affinity compounds based on their binding energies. To understand the stability of these complexes, molecular dynamic simulations were executed and the trajectory analysis exposed that the NS3 helicase-ellagic acid complex possesses greater stability than the other two complexes such as NS3 helicase-hypericin and NS3 helicase-pentagalloylglucose. The ADMET property prediction of these compounds resulted in nontoxicity and noncarcinogenicity.

## 1. Introduction

Zika virus (ZIKV) is an arbovirus related to the family of *Flaviviridae* [1]. It is a positive sense RNA, which is a constituent of the genus *Flavivirus.* This genus includes other viruses such as tick-borne encephalitis, yellow fever, tick-borne encephalitis, dengue, West Nile, Langat, St. Louis encephalitis, Modoc, Powassan, Japanese encephalitis, and Rio Bravo viruses [2]. The genome length of ZIKV is 10,794 kb, where the RNA has two noncoding regions such as 39 and 59 with a long open reading frame. ZIKV differs from other flaviviruses through the glycosylation spots located on the surface of the virus [3]. The ZIKV genome has three structural proteins and seven nonstructural proteins, which mediate the replication of the genome [4]. Here, the viral transmission follows the sylvatic cycle (nonhuman primate's cycle), where the virus is disseminated by the bite of mosquitoes, namely, *Aedes albopictus*, *Aedes aegypti*, *Aedes africanus*, and *Aedes hensilli*, infected with ZIKV [5]. It can affect cells like dermal fibroblasts, skin keratinocytes, and dendritic cells. *In vitro* studies reveal that these cells demonstrated an increased level of infection after 24 to 48 hours of viral entry [6]. After biting an infected person, the mosquitoes consume the blood with ZIKV that initiate replication in the midgut epithelial cells and transfer to the salivary gland. After the incubation time of 10 days, the saliva is infected, which starts spreading by biting the other human. Through clathrin-mediated endocytosis, the viruses enter the host cell. When ZIKV enters the host skin, initially it affects the dermal fibroblasts that serve as a receptor for the viral attachment. Then, the membrane of the virus fuses with the endosomal membrane and would be delivered into the mammalian cells [7]. After completion of the replication, the virus gets multiplied and affects the lymph nodes and nervous system [8]. It also spreads through sexual intercourse and from the mother to the fetus through the placenta at the time of pregnancy [9]. These situations cause microcephaly and congenital Zika syndrome in the fetus. Neurological problems like Guillain-Barre syndrome (in adults—autoimmune disease), myelitis, and neuropathy were also reported in ZIKV infection [10]. The symptoms include mild rash, fever, joint and muscle pain, conjunctivitis, malaise, or headache, including symptomatic cases. It has been reported that terrorists are responsible for the vigorous transmission of ZIKV worldwide in around 60 nations [11]. In adults, the infection is associated with other defects such as thrombocytopenia, meningitis, multiorgan failure, and encephalitis. Mortality has been reported with sickle cell disease in children and cancer in adults. The persistence of ZIKV in the eye leads to the replication of the virus and causes maculopathy, uveitis, and conjunctivitis, which may tend to blindness in many cases [12]. Temperature is the greatest driver of vector-borne disease transmission; the mosquito transmission decreases with temperature. When the disease transmission occurs at 29°C, it was controlled under a cool temperature of <22°C. Also, the effectiveness of genome replication was reduced at cool temperatures [13]. The extensive outbreaks of ZIKV and its neurological defects have created a greater public health concern worldwide. There is an urgent need to identify the most promising inhibitor against ZIKV. Most of the existing antivirals are reported to have side effects. Hence, our current study exploited plant-derived phytochemicals against ZIKV, which has less toxicity as well as less chance to develop resistance. Usually, these plant-derived compounds were exploited to treat many diseases. Also, these plant compounds can inhibit replication. Alkaloids such as cepharanthine, fangchinoline, and tetrandrine isolated from *Stephania tetrandra S. Moore* have the ability to conceal the viral protein expression and thereby inhibit the human coronavirus OC43 replication in MRC-5 cells [14]. Synthesized or plant-derived quinolone alkaloids inhibit the activity of type II topoisomerase which leads to the inhibition of DNA replication [15]. Hence, our study has targeted the nonstructural ZIKV NS3 helicase, a crucial enzyme that participates in RNA unwinding of the replication process. The helicases of the Flavivirus are also informed to contribute to other vital roles like the splicing of pre-mRNA, ribosome biogenesis, export and degradation of RNA, maturation of RNA, and translation process. This NS3 helicase is a member of the superfamily SF2, which has a close identity with the Murray Valley encephalitis virus, dengue virus 2 (DENV2), and DENV4. The N terminal region of the protein possesses the activity of protease, and the C terminal region possesses the activity of helicase. Though the helicases of the Flavivirus family have conserved active site regions, ZIKV NS3 helicase has different RNA binding modes and motor domain movements [16]. All these crucial roles of ZIKV NS3 helicase inspired us to screen plant-derived phytochemicals. Various studies have been conducted to screen inhibitors against ZIKV; they used crucial targets such as NS3 helicase [17], NS2B/NS3 protease [18] [19, 20], and glycoprotein (gp) E [21]. The current study involves the use of docking protocols to analyze the binding mode of 25 plant compounds with the ZIKV NS3 helicase, molecular dynamic studies were further focused to analyze the protein-ligand complex stability, and finally, the oral bioavailability of the best binding energy (BE) ligands was also predicted. The overall study reveals the favorable BE, stability, and bioavailability of the plant compound ellagic acid. Our study will be helpful to the scientist who is involved in the inhibitor design for the ZIKV.

## 2. Materials and Methods

### 2.1. Preparation of the Target

**Figure 1:**
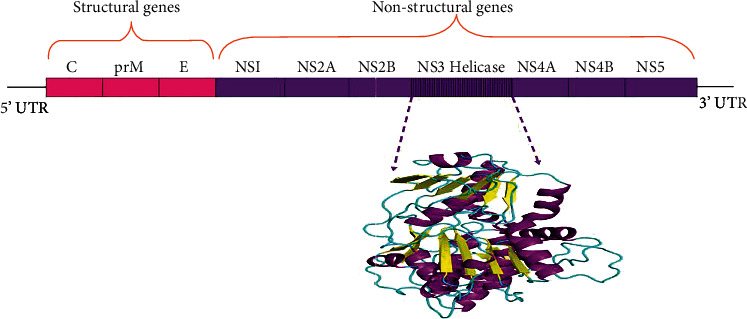
The ZIKV genome comprises structural and nonstructural genes. The location of the nonstructural protein NS3 helicase (PDB: 5JRZ) was highlighted.

**Figure 2:**
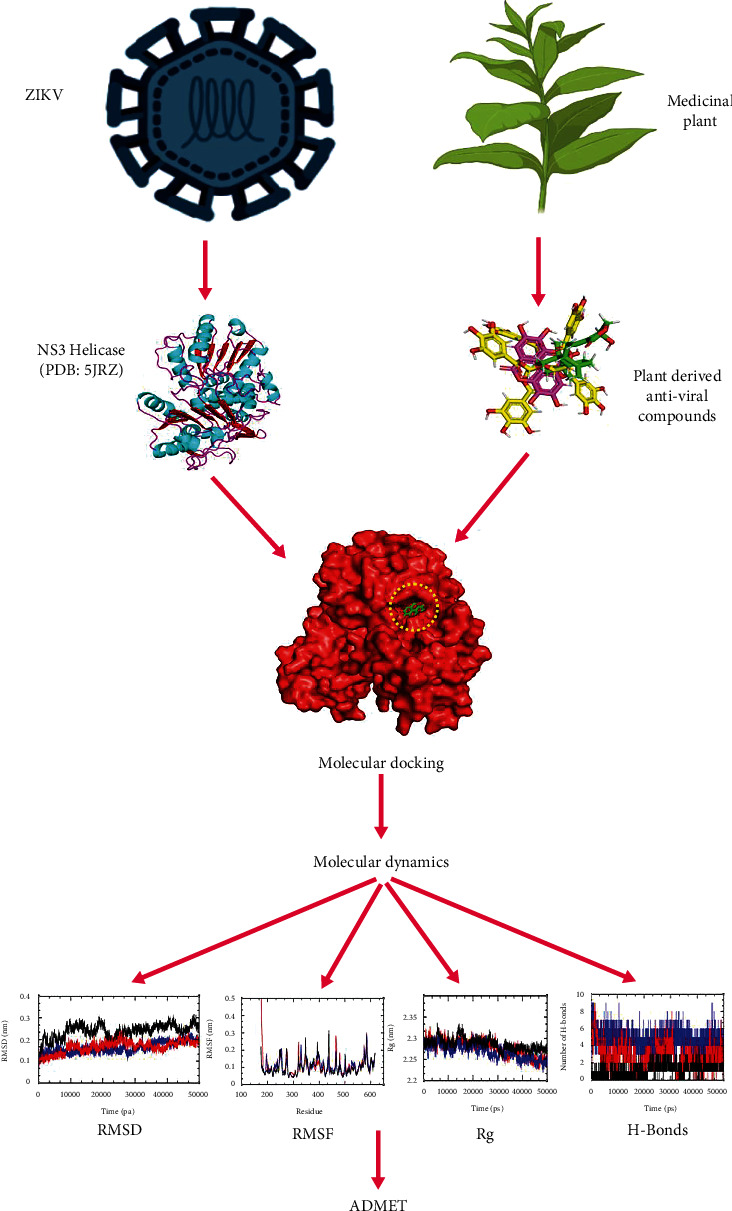
The stepwise workflow of the complete study.

The ZIKV NS3 helicase structure was obtained through the database Protein Data Bank (PDB: 5JRZ) [22, 23]. It is a monomer with a resolution of 1.62 Å. The X-ray diffraction technique was used to determine the crystal structure (Figure 1). It has a single chain with an amino acid length of 449. Nonresidue atoms coordinated with the crystal structure were removed. The missing residues and the atoms were modeled through the MODELLER 9.25 server [24].  Figure 2 depicts the study's whole workflow. The figure was drawn with the help of the BioRender server [25].

### 2.2. Preparation of Active Sites

The amino acid information of the ATP binding site region in the ZIKV NS3 helicase (PDB: 5JRZ) structure was recovered from the literature [22].

### 2.3. Ligand Preparation

A total of twenty-five plant compounds with antiviral activity were collected from various studies [26–49], and their 3D structures were collected from the PubChem database [50]: apigenin (ID: 5280443), baicalein (ID: 5281605), berberine (ID: 2353), betulin (ID: 72326), chebulagic acid (ID: 442674), curcumin (ID: 969516), ellagic acid (ID: 5281855), epigallocatechin gallate (ID: 65064), fisetin (ID: 5281614), geraniin (ID: 3001497), glycyrrhizic acid (ID: 14982), hypericin (ID: 3663), hyperoside (ID: 5281643), kaempferol (ID: 5280863), lupeol (ID: 259846), mimusopic acid (ID: 6712545), mulberroside C (ID: 190453), myricetin (ID: 5281672), neoandrographolide (ID: 9848024), pentagalloylglucose (ID: 65238), piperine (ID: 638024), quercetin (ID: 5280343), rosmarinic acid (ID: 5281792), rutin (ID: 5280805), and torvoside (ID: 11018078). Also, N-(3-acetylphenyl) morpholine-4-carboxamide (ID: 671267), 2-(2-acetamidophenyl) acetic acid (ID: 14622178), and 2-(4-acetamidophenyl) acetamide (ID: 25862032) were obtained from the PubChem database.

### 2.4. Drug-Likeness Property Prediction for Phytochemicals

The drug candidates usually act upon the cellular targets; the molecule binds to the target and changes the cellular machinery and produces therapeutic action. A molecule before producing the pharmacodynamics effect on the human body should travel from the entry point to the active site, which is referred to as the pharmacokinetic properties. It was defined by the terms like absorption, distribution, metabolism, and excretion. To predict such pharmacokinetic properties of the drugs, Lipinski and coworkers have established a set of rules known as “Lipinski's rule of five.” This rule states that the H-bond donors should be below 5, H-bond acceptors should not exceed 10, mlogP value should be below 5, and molecular weight should be below 500 Daltons [51]. These rules filter the compounds and make the drug candidates orally available. To analyze the oral bioavailability of the plant-derived compounds, the drug-likeness property of the compounds was predicted through the Molinspiration server [52].

### 2.5. Molecular Docking Studies

The interaction of small molecules with the target protein can be understood by molecular docking studies. For our study, we have utilized the AutoDock Vina, an open-source tool [53]. The structures of target NS3 helicase (PDB: 5JRZ) and 25 plant compounds were submitted as the input file. The protonation state of the amino acid side chains was predicted through the online server PropKa [54]. The stereochemistry of all the compounds was analyzed properly. Through the SWISS-PdbViewer, all the ligands were energy minimized [55]. SWISS-PdbViewer employs the steepest descent energy minimization through the GROMOS96 force field [56]. The grid size was fixed as 80 × 80 × 80 with 0.375 Å spacing. Also, grid center XYZ coordinates were set as 39.629, 27.127, and 51.699, respectively. AutoDock Vina uses iterated local search global optimizer (a genetic algorithm with local gradient optimization), a stochastic global optimization algorithm, and hybrid scoring functions (a combination of empirical and knowledge-based scoring functions) [57]. AutoDock Vina produces the ten best binding modes for each run. The negative binding energy designates the prediction of ligand binding to a target protein. The higher negative value of the binding energy indicates the favorable interaction between the protein and ligand.

### 2.6. Molecular Dynamic Simulations

The simulation studies were executed to explore the stability of the selected bound complexes. From the molecular docking results, the three best BE complexes, NS3 helicase-ellagic acid, NS3 helicase-hypericin, and NS3 helicase-pentagalloylglucose were subjected to molecular dynamic simulations for 100 ns through GROMACS package in the Ubuntu environment [58] using Intel Xeon W-1270, 8 core, and 16 threaded processors. The complexes were located in the cubic box, and using transferable intermolecular Potential 3P (TIP3P) water, the system was solvated. The topology of the protein was produced through the Chemistry at Harvard Macromolecular Mechanics 36 (CHARMM36) force field, and for ligand, it was created by CHARMM General Force Field (CGenFF) 4.4 version [59]. The whole system was neutralized by adding the Na^+^ or Cl^−^ ions. Energy minimization of the complete system was carried out using the steepest descent algorithm. Next, two sets of equilibration were performed, one is NVT (canonical ensemble) run for 0.1 ns and another one is NPT (isothermal–isobaric ensemble) run for 0.1 ns. The bond length of the protein was restrained using the LINCS algorithm [60], and the Particle Mesh Ewald method was used to measure the electrostatic interactions. Finally, the production run of 100 ns was planned with 1.0 bar pressure and 300 K temperature. The trajectory analysis was made to produce different plots like Root Mean Square Deviation (RMSD), Root Mean Square Fluctuation (RMSF), Radius of Gyration (Rg), H-bond interactions, and Molecular Mechanics Poisson-Boltzmann Surface Area (MMPBSA) calculation of binding free energy which were accomplished to understand the stability of the complexes.

### 2.7. In Silico Absorption, Distribution, Metabolism, Excretion, and Toxicity (ADMET) Prediction

In the current study, we have used the admetSAR online tool for the ADME predictions [61]. An ADMET structure-activity relationship database has ADMET-related properties collected from the literature. It has 210000 data points for more than 96000 unique compounds. In absorption, BBB permeability, HIA, Caco-2 absorption, P-gp (drug transporter) inhibitor and substrate, and renal OCTs were analyzed. In metabolism, the cytochrome (CYP) P450 substrate 2C9, 2D6, 3A4 and CYP P450 inhibitors 1A2, 2C9, 2C19, 2D6, and 3A4 were analyzed. Also, toxicity and carcinogenicity of the small molecules were predicted by the admetSAR server.

## 3. Results

A protein-ligand docking and molecular dynamic simulations were conducted to locate the potential NS3 helicase inhibitors.

### 3.1. Active Site Prediction

**Figure 3:**
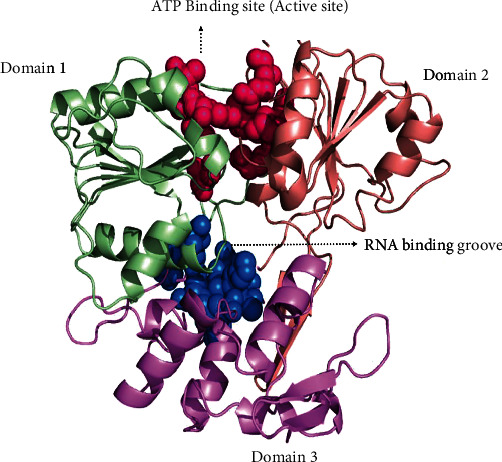
Active sites of the ZIKV NS3 helicase (PDB: 5JRZ). ATP binding site is highlighted in pink.

According to Jain et al. (2016), the ZIKV NS3 helicase (PDB: 5JRZ) structure has three domains (domains 1, 2, and 3) with more or less similar sizes and two binding sites such as ATP binding site located between domains 1 and 2 and the RNA binding groove located between 1 and 3 (Figure 3). For the current study, we have retrieved the ATP binding site amino acids (Ala416, Glu231, Arg459, Glu286, Gln455, Arg202, Arg462, Pro196, and Lys200) to carry out the docking studies [22].

### 3.2. Drug-Likeness Property Prediction of Plant Compounds

All the 25 plant-derived antiviral compounds collected from the literature (Figure S1) were subjected to drug-likeness property prediction; out of which, a few compounds such as chebulagic acid, geraniin, glycyrrhizic acid, pentagalloylglucose, rutin, and torvoside showed 3 violations, where their molecular weight, hydrogen bond donor (HBD), and hydrogen bond acceptor (HBA) were found higher than the limited values. Lipinski's rule of the five compounds violated only two rules. Similarly, the compounds epigallocatechin gallate and hyperoside had 2 violations. Here, we have observed several HBA and HBD counts. Similarly, betulin, lupeol, and myricetin had one violation, which was due to excess octanol partition coefficient values of botulin and lupeol, respectively; myricetin HBD count was found to be higher than the normal value (Table 1).

### 3.3. Molecular Docking Studies

Docking of the 25 plant compounds was carried out through the AutoDock Vina. The binding energies resulted in the range of -7.1 kcal/mol to -9.9 kcal/mol for the plant compounds against nonstructural protein NS3 helicase (PDB: 5JRZ) (Table 2). Here, favorable binding energy was observed for ellagic acid. The compounds ellagic acid, hypericin, pentagalloylglucose, epigallocatechin gallate, rutin, and glycyrrhizic acid fell within the binding energy ranges from -9.1 kcal/mol to -9.9 kcal/mol. Compounds such as mulberroside, myricetin, quercetin, baicalein, fisetin, torvoside, hyperoside, kaempferol, lupeol, neoandrographolide, and rosmarinic acid showed their binding energies within -8.0 kcal/mol to -8.9 kcal/mol. The remaining compounds like apigenin, chebulagic acid, mimusopic acid, berberine, betulin, curcumin, geraniin, and piperine fall within the binding energies between -7.1 kcal/mol and -7.9 kcal/mol.

The binding free energy was contributed by the interactions of electrostatic and nonelectrostatic forces. Here, the contribution of both the interactions was quantified by Coulomb's potential and Lennard Jones potential, respectively. The contribution of each force is based on the charge and shape complementarity of the protein-ligand complex.

**Figure 4:**
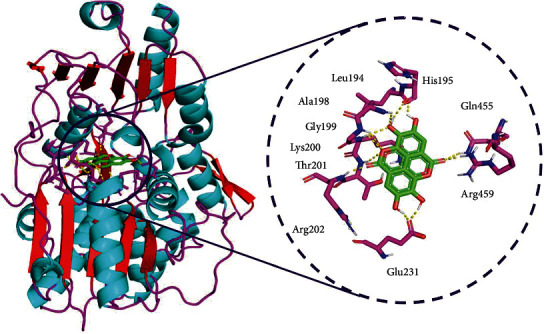
Binding mode of ZIKV NS3 helicase (PDB: 5JRZ) with ellagic acid obtained through AutoDock Vina docking. A close view represents the amino acid interaction between the NS3 helicase (PDB: 5JRZ) and ellagic acid.

From Table 3, the most favorable interacting complexes were selected. Here, NS3 helicase-ellagic acid complex was ranked first as the interaction energy was observed to be -9.9 kcal/mol. It exhibited H-bond contacts with nine amino acids of Arg459 (bond length, 2.1 Å), Gln455 (2.4 Å), Glu231 (1.8 Å & 1.9 Å), Arg202 (2.1 Å), Thr201 (2.5 Å), Gly199 (2.8 Å), Ala198 (2.8 Å), Leu194 (2.0 Å), and His195 (2.1 Å) and hydrophobic interactions found with the amino acids of Arg776, Thr790, Leu858, Cys775, Leu777, Lys745, Asp855, Thr854, Met766, Leu844, Val726, Met793, Cys797, Ala743, Leu792, Leu718, Met1002, and Gly796 (Figure 4). These intermolecular H-bond and hydrophobic interactions are stabilizing the ligands in the binding sites of the NS3 helicase protein.

**Figure 5:**
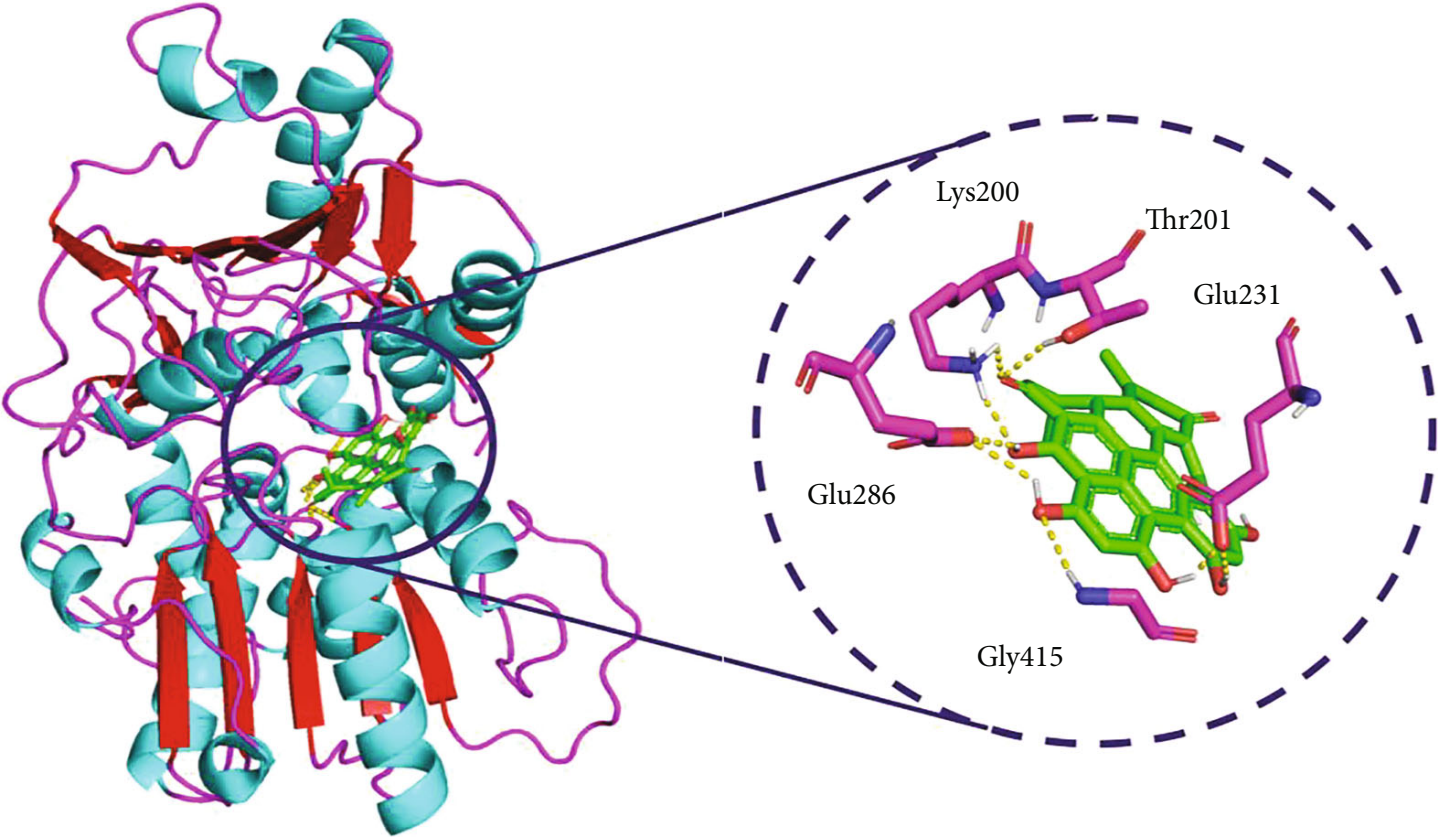
Binding mode of ZIKV NS3 helicase (PDB: 5JRZ) with hypericin obtained through AutoDock Vina docking. A close view represents the amino acid interaction between the NS3 helicase (PDB: 5JRZ) and hypericin.

Hypericin was ranked second with -9.8 kcal/mol of binding energy and 5 amino acid interactions of Glu286 (2.7 and 3.4), Gly415 (2.6), Glu231, Thr201 (2.4), and Lys200 (2.2 and 2.3) and hydrophobic interactions of Gly415, Glu231, Ala416, Arg462, Arg202, Gly197, Asn417, and Gly199 (Figure 5).

**Figure 6:**
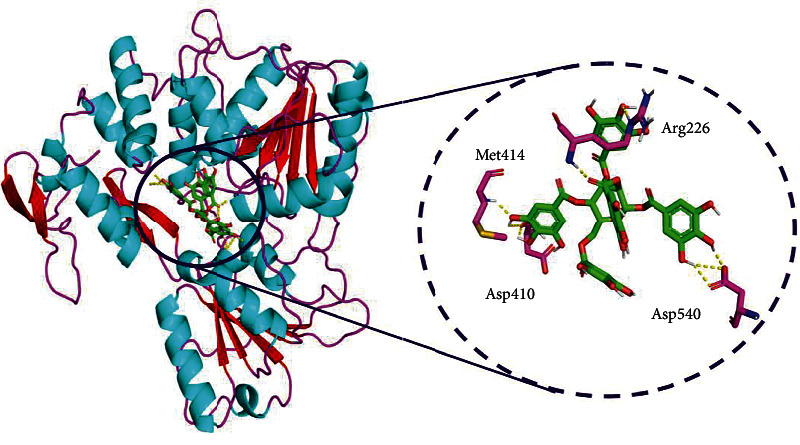
Binding mode of ZIKV NS3 helicase (PDB: 5JRZ) with pentagalloylglucose obtained through AutoDock Vina docking. A close view represents the amino acid interaction between the NS3 helicase (PDB: 5JRZ) and pentagalloylglucose.

Similarly, pentagalloylglucose was positioned third in the list with the binding energy of 9.5 kcal/mol and stabilized by 4 H-bonds with amino acids such as Asp410 (1.9 and 2.6), Met414 (2.0), Asp540 (1.9, 2.6, and 2.1), and Arg226 (2.0 and 2.1). It also displayed ten hydrophobic interactions with Asp291, Val227, Glu413, Arg388, Thr225, Ile411, Phe391, Cys262, Ala264, and Phe289 (Figure 6).

H-bonds are more important in defining the inhibitor's activity against the target protein and in ensuring its stability with the protein. Here, the active site (ATP binding site) amino acids of NS3 helicases, such as Arg202, Arg459, Glu231, and Gln455, are involved in the interaction with ellagic acid; likewise, Glu286, Glu231, and lys200 amino acids were connected with the hypericin. None of the active site amino acids are found in the H-bond interactions between the NS3 helicase and pentagalloylglucose. Among the active site residues, Glu231 was found closely interacted with ellagic acid through 2 H-bonds with distances of 1.8 Å and 1.9 Å.

### 3.4. Molecular Dynamic Simulation Studies

The structural stability of the best binding energy complexes, NS3 helicase-ellagic acid, NS3 helicase-hypericin, and NS3 helicase-pentagalloylglucose complexes was analyzed by conducting molecular dynamic simulations for 100 ns, and the RMSD, RMSF, Rg, H-bond interactions, and MMPBSA calculation of binding free energy were analyzed.

#### 3.4.1. RMSD

**Figure 7:**
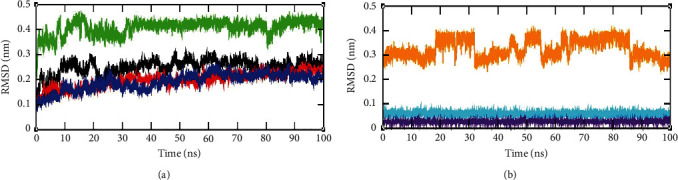
RMSD plot obtained through GROMACS trajectory analysis. (a) RMSD plot of protein backbone for complexes such as NS3 helicase (PDB: 5JRZ)-ellagic acid (blue), NS3 helicase (PDB: 5JRZ)-hypericin (red), NS3 helicase (PDB: 5JRZ)-pentagalloylglucose (black), and unbound NS3 helicase (PDB: 5JRZ) (green). (b) RMSD plot for ligand atoms ellagic acid (indigo), hypericin (cyan), and pentagalloylglucose (orange).

The RMSD is a measure of the difference between the initial structural confirmations of the protein backbone to its final position. The stability of the protein related to the structural conformation can be estimated by the deviations produced after the simulations. The RMSD plot for the C*α* backbone atoms of the complexes NS3 helicase-ellagic acid, NS3 helicase-hypericin, and NS3 helicase-pentagalloylglucose was depicted in Figure 7(a). As shown in the figure, NS3 helicase-ellagic acid (blue) and NS3 helicase-hypericin (red) displayed slight variations in their RMSD values. At the beginning of the simulations, NS3 helicase-ellagic acid complex had the RMSD of 0.10 nm, which reached 0.12 nm until 10 ns. Similarly, the NS3 helicase-hypericin complex showed an RMSD value of 0.13 nm in the beginning and reached 0.15 nm till 10 ns; after which, both the complexes started showing deviation in their RMSD values. After 30 ns of simulation, both the complexes attained stability at 0.20 nm and 0.23 nm. Likewise, the NS3 helicase-pentagalloylglucose complex (black) showed fluctuation in the beginning and reached 0.25 nm after 10 ns. Further, as illustrated in the figure, we observed a fall in the RMSD value after 20 ns and attain stability at 0.27 nm. The unbound NS3 helicase has shown a stable RMSD of 0.42 nm after 30 ns (green). The overall comparison of the RMSDs of the three different complexes and unbound protein indicates the lower stability of the ellagic acid.  Figure 7(b) depicts the RMSDs of the ligands ellagic acid (indigo), hypericin (cyan), and pentagalloylglucose (orange) after 100 ns. The ligands ellagic acid, hypericin, and pentagalloylglucose found their stability state at 0.02 nm, 0.06 nm, and 0.3 nm, respectively. The comparison of the RMSDs of the unbound NS3 helicase and free ligands with bound protein-ligand complex unfolds the impact of ligands binding with the NS3 helicase.

#### 3.4.2. RMSF

**Figure 8:**
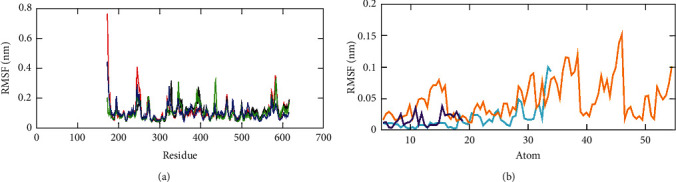
RMSF plot obtained through GROMACS trajectory analysis. (a) RMSF per residue plot for protein-ligand complexes, NS3 helicase (PDB: 5JRZ)-ellagic acid (blue), NS3 helicase (PDB: 5JRZ)-hypericin (red), NS3 helicase (PDB: 5JRZ)-pentagalloylglucose (black), and unbound NS3 helicase (PDB: 5JRZ) (green). (b) RMSF plot for ligand atoms ellagic acid (indigo), hypericin (cyan), and pentagalloylglucose (orange).

Fluctuations of each amino acid in the NS3 helicase (PDB: 5JRZ), when bound to compounds such as ellagic acid, hypericin, and pentagalloylglucose, are illustrated in Figure 8(a). The NS3 helicase and ellagic acid exhibited their amino acid interactions at Arg459, Gln455, Glu231, Arg202, Thr201, Gly199, Ala198, Leu194, and His195. Likewise, NS3 helicase-hypericin interacted with the amino acids Glu286, Gly415, Glu231, Thr201, and Lys200. Also, NS3 helicase-pentagalloylglucose interacted with Asp410, Met414, Asp540, and Arg226, respectively. The unbound NS3 helicase has shown the greater fluctuation (green) when compared to the other three complexes and the least fluctuation was observed for NS3 helicase-ellagic acid complex (blue). These fluctuation ranges revealed the higher stability of the NS3 helicase-ellagic acid complex than that of the other two complexes NS3 helicase-hypericin and NS3 helicase-pentagalloylglucose.  Figure 8(b) depicts the RMSF values for three ligands, ellagic acid (indigo), hypericin (cyan), and pentagalloylglucose (orange). The highest fluctuation was observed for pentagalloylglucose, followed by hypericin and ellagic acid. Hence, the binding of phytochemicals with NS3 helicase has a greater impact on the rigidity of the protein-ligand complex.

#### 3.4.3. Rg

**Figure 9:**
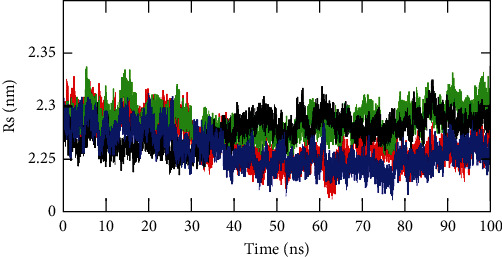
Rg plot for C*α* atoms obtained through GROMACS trajectory analysis. Rg plot for NS3 helicase (PDB: 5JRZ)-ellagic acid (blue), NS3 helicase (PDB: 5JRZ)-hypericin (red), NS3 helicase (PDB: 5JRZ)-pentagalloylglucose (black), and unbound NS3 helicase (PDB: 5JRZ) (green).

The calculation of Rg revealed the compactness of the protein after binding to the ligand.  Figure 9 shows the Rg values of the complexes NS3 helicase-ellagic acid (blue), NS3 helicase-hypericin (red), and NS3 helicase-pentagalloylglucose (black) and unbound NS3 helicase (green). From the beginning of the simulation to 30 ns, all the three complexes attained stability at the Rg values of 2.29 nm. Later, a decrease in Rg value was observed for all the three complexes after 30 ns. Both NS3 helicase-ellagic acid and NS3 helicase-hypericin were observed to gain stability at 2.24 nm. The lack of variations in the complex revealed the rigidity of the NS3 helicase protein after binding with the ligands ellagic acid and hypericin. As illustrated in the figure, the complex NS3 helicase-pentagalloylglucose gained stability at 2.27 nm and unbound NS3 helicase gained stability at 0.29 nm. The variation in the Rg values between these complexes and free protein revealed the rigidity of the complex after binding to the ligands.

#### 3.4.4. H-Bond Interactions of Protein-Ligand Complexes

**Figure 10:**
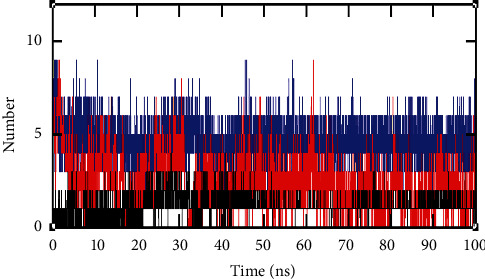
H-bond interactions were obtained through GROMACS trajectory analysis. Number of H-bonds formed between the NS3 helicase (PDB: 5JRZ)-ellagic acid (blue), NS3 helicase (PDB: 5JRZ)-hypericin (red), and NS3 helicase (PDB: 5JRZ)-pentagalloylglucose (black).

The H-bond formation between the protein and ligands generates specificity and directionality, which is the basic aspect of molecular recognition. The HBD and HBA share their energy in the binding site region. Also, for further stability of the complex, it must be complemented with the van der Waals interactions because of the shape complementarity. After the completion of 100 ns of MD simulations, the H-bond interaction between the complexes NS3 helicase-ellagic acid, NS3 helicase-hypericin, and NS3 helicase-pentagalloylglucose was analyzed. As depicted in Figure 10, the NS3 helicase-ellagic acid complex (blue) formed 7 strong H-bonds throughout the simulation. The NS3 helicase-hypericin complex (red) with 5 strong H-bond formations and NS3 helicase-pentagalloylglucose (black) with 3 H-bond formations were observed throughout the simulations. These H-bond formations stabilized the protein-ligand complexes.

#### 3.4.5. MMPBSA Calculation of Binding Free Energy

The binding free energy for the phytochemicals ellagic acid, hypericin, and pentagalloylglucose with the target NS3 helicase (PDB: 5JRZ) was calculated by the MMPBSA technique. The total binding free energy is the combination of van der Waals energy, electrostatic energy, polar solvation energy, and solvent-accessible surface area (SASA). The van der Waals energy, electrostatic energy, and SASA contribute negative energy values, and polar solvation energy contributes positive energy values to the total BE. The comparison of three binding energies revealed the highest total BE of ellagic acid (−346.4 ± 7.1 kJ/mol) than that of the other two complexes, which indicated the strong interaction of ellagic acid with the ZIKV NS3 helicase (PDB: 5JRZ). Here, the van der Waals energy contributed more than the electrostatic energy (Table 4).

### 3.5. In Silico ADMET Prediction

Drug designing and development is highly a complex, time-consuming, and costlier process which has increased attrition rates. The development of in silico ADMET predictions has reduced the failure rate in recent years. ADMET prediction is an essential component in the analysis of the efficacy and toxicity of the drug. It also determines the administration dose, route, and frequency. The physicochemical property of the compound candidate may affect the ADMET properties. Hence, the assessment of ADMET property is vital for the successful development of drugs. The initial evaluations of pharmacokinetic and toxic properties are essential to avoid compounds with ADME problems, which helps the scientist to prioritize particular compounds to synthesize and evaluate. Prediction of ADMET properties of these plant-derived compounds may save the time and cost of drug discovery. The blood-brain barrier (BBB) controls the flow of chemicals into the brain. The role of BBB is to separate the flow of blood from the central nervous system. This prevents harmful substances to enter the brain tissues. BBB controls the movement of external compounds to preserve the central nervous system at a steady state. Here, ellagic acid and pentagalloylglucose may cross the BBB (BBB_positive_) and hypericin may not cross the BBB (BBB_negative_). The phytochemicals ellagic acid and hypericin may get absorbed by the intestine (HIA_positive_) and pentagalloylglucose may not be absorbed by the intestine (HIA_negative_). The Caco-2 permeability was analyzed to identify the intestinal absorption of the drugs; the results showed the Caco-2 permeability of hypericin (Caco2_positive_) and nonpermeability of ellagic acid and pentagalloylglucose (Table 5). The P-gp enzyme acts as a drug transporter, which controls the uptake and efflux of a wide variety of drugs. It also serves as an efflux pump. P-gp facilitates drug-drug interactions. The substrates of P-gp can also act as an inhibitor of the enzyme; inhibition of the enzyme results in greater drug bioavailability. Here, these three plant compounds, namely, ellagic acid, hypericin, and pentagalloylglucose, function as the substrate for P-gp. Similarly, cytochrome P450 enzymes such as 2D6, 2C9, and 3A4, hypericin, and the ellagic acid act as a nonsubstrate, and pentagalloylglucose serves as a substrate. The AMES toxicity and carcinogenic test revealed the nontoxicity and noncarcinogenic effects of ellagic acid, hypericin, and pentagalloylglucose.

## 4. Discussion

ZIKV is one of the increasingly reported viral pathogens that sharply spreads beyond geographical borders. Since 2015, there are an increased number of microcephaly and Guillain-Barre syndrome incidences that have been observed worldwide. Specific vaccines or proper treatment option was not available to treat ZIKV till today. Few medications have been prescribed to release pain and fever related to the viral infection. Also, engineering methods were applied to identify potential peptides that penetrate the brain and cure infections. Apart from this, Ayurveda treatment is the most appropriate method to treat infections, which comprises only natural constituents and has no side effects. Hence, in our study, the molecular modeling protocols opted to identify natural phytochemicals as the potential inhibitors for ZIKV. Here, an active site-specific docking procedure was implemented and the active site residues were retrieved from the latest literature [22]. In the NS3 helicase (PDB: 5JRZ) structure, both RNA and ATP binding regions possess polar and hydrophobic characteristics and were suitable for high throughput screening; we utilized ATP binding sites for our study. A similar analysis conducted by Badshah et al. in 2019, which also utilized ATP binding site to carry out molecular docking for NS3 helicase against 1,4-benzothiazine derivatives to find out potential inhibitors for ZIKV [17]. According to “Lipinski's rule of five,” the oral bioavailability of the natural constituents was assessed. This rule permits compounds with 2 violations. According to Benet et al.'s study in 2016, the rule of 5 is not applicable for natural compounds and natural compound derivatives [62]. To evaluate the nature of the compounds, the drug-likeness property prediction was carried out. Many computational studies report the use of screening techniques to identify potential inhibitors for various disease conditions. Kumari and Subbarao (2020) used FDA*-*approved drugs, natural products, and phytochemicals to screen against glutamine synthetase of *Mycobacterium tuberculosis*, an important enzyme of cell wall synthesis and nitrogen metabolism [63]. Sen et al. (2020) applied a structure-based virtual screening procedure to identify inhibitors for Severe Acute Respiratory Syndrome Coronaviruses 2 (SARS-CoV-2) [64]. They used phytochemicals retrieved from ginger, garlic, onion, peppermint, fenugreek, and chili to screen against the targets spike and main protease. Similarly, Gahlawat et al. (2020) screened inhibitors for the target main protease of SARS CoV-2; they used main protease inhibitors, natural products, and FDA-approved drugs [65]. The active site helps ligands to create enough contact sites to produce excellent interaction with the target protein by maintaining appropriate and desirable catalytic microenvironments [66–69]. In the ATP binding site of the NS3 helicase, the amino acids Glu231, Arg459, Gln455, Arg202, Glu286, Glu231, and Lys200 play a key role in the interaction with the ligands ellagic acid, hypericin, and pentagalloylglucose. From the docking results' best binding energy compounds, ellagic acid (-9.5 kcal/mol), hypericin (-9.2 kcal/mol), and pentagalloylglucose (9.0 kcal/mol) were finally selected as the potential compounds. These docking results mimic the experimental study conducted by Acquadro et al. in 2020, which revealed the antiviral activity of leaf ethanolic extract of *Punica granatum* and ellagic acid against ZIKV [70]. The activity screening was carried out against the MR766 and HPF2013 strains of ZIKV. The study concluded that the compounds and the extracts were found to be active against the ZIKV; in particular, ellagic acid has shown EC_50_ values of 30.86 *μ*M and 46.23 *μ*M for the strains MR766 and HPF2013, respectively. Another study revealed the antihuman rhinovirus (HRV) activity of ellagic acid isolated from the leaves of *Lagerstroemia speciosa*. The different strains of HRVs, namely, HRV-2, HRV-3, and HRV-4, have a 50% inhibitory concentration range of 38 *μ*g/mL, 31 *μ*g/mL, and 29 *μ*g/mL, respectively, which was higher than that of the ribavirin drug. It also suggested that 50 *μ*g/mL of ellagic acid inhibited viral replication by aiming at cellular components [31]. A randomized trial on ellagic acid and high-risk human papillomavirus- (HPV-) related low squamous intraepithelial lesion in women revealed a 74% HPV clearance in a group supplemented with 16 mg ellagic acid along with 100 mg of *Annona muricata* than the placebo group [71]. To find an inhibitor for Ebola virus cell entry, a high throughput assay was conducted with the extracts of 128 traditional medicine, which revealed that the crude extract and the compounds gallic acid and ellagic acid isolated from *Rhodiola rosea* were found to be effective against Ebola virus [72]. Ellagic acid showed favorable activity against many viruses. Hypericin, plant quinine, inactivates the human immunodeficiency virus type 1 (HIV-1), Sindbis virus, and murine cytomegalovirus [73]. Similarly, the pentagalloylglucose was also found to be effective against the influenza A virus, as it exhibited virus-induced hemagglutination in chicken red blood cells. Without affecting the nuclear transport of nucleoprotein or protein synthesis, pentagalloylglucose reduced the nucleoprotein accumulation in the plasma membrane during the replication cycle [44]. Jin et al.'s study in 2016 showcased the antiherpes simplex virus (HSV) activity of pentagalloylglucose, which slows down the process of nuclear transport in HSV-1 by blocking the upregulation of dynein [74]. Also, it affected the process of nucleocapsid egress in HSV-1. From the literature, it is evident that the plant compounds ellagic acid, hypericin, and pentagalloylglucose possess antiviral activity.

To understand the stability of the selected compounds, molecular dynamic studies were carried out for the complexes NS3 helicase-ellagic acid, NS3 helicase-hypericin, and NS3 helicase-pentagalloylglucose complexes, which revealed the higher stability of the complex; NS3 helicase-ellagic acid than the NS3 helicase-hypericin confirmed the nontoxicity and noncarcinogenicity of the compounds.

Medicinal plants serve as the most promising basis of natural antiviral substances against ZIKV. Bioactive substances isolated from medicinal plants have established their value against many life-threatening diseases like malaria, cancer, diabetes, heart disease, and Alzheimer's diseases [75]. In the current study, a few antiviral plant compounds were screened against the NS3 helicase of ZIKV through molecular docking and molecular dynamic techniques and found ellagic acid as the more potential compound against ZIKV. Further, preclinical and clinical studies must be carried out to confirm the antiviral activity of ellagic acid against ZIKV.

## 5. Conclusion

The mosquito-transmitted ZIKV disease has now emerged as a public health threat worldwide due to its ability to cause neural infections. To identify potential inhibitors for ZIKV, we have screened medicinal plant-derived antiviral compounds against the nonstructural protein NS3 helicase of ZIKV. We conducted molecular docking and molecular dynamic studies and found ellagic acid as the most favorable binding energy compound against ZIKV NS3 helicase. Further clinical studies must be conducted on ellagic acid to ensure its antiviral activity against ZIKV.

## Figures and Tables

**Table 1:** Molinspiration predicted drug-likeness properties of the phytocompounds.

S. no.	Phytocompounds	mlogP (<5)	MW (<500)	HBA count (<10)	HBD count (<5)	RB count (<10)	No. of violations
1	Apigenin	2.46	270.24	5	3	1	0
2	Baicalein	2.68	270.24	5	3	1	0
3	Berberine	0.20	336.37	5	0	2	0
4	Betulin	7.16	442.73	2	2	2	1
5	Chebulagic acid	0.07	954.66	27	13	5	3
6	Curcumin	2.30	368.38	6	2	8	0
7	Ellagic acid	0.94	302.19	8	4	0	0
8	Epigallocatechin gallate	2.25	458.38	11	8	4	2
9	Fisetin	1.97	286.24	6	4	1	0
10	Geraniin	-0.78	952.65	27	14	3	3
11	Glycyrrhizic acid	1.97	822.94	16	8	7	3
12	Hypericin	5.77	504.45	8	6	3	0
13	Hyperoside	-0.36	464.38	12	8	4	2
14	Kaempferol	2.17	286.24	6	4	1	0
15	Lupeol	8.29	426.73	1	1	1	1
16	Mimusopic acid	4.26	486.69	5	4	2	0
17	Mulberroside C	1.96	458.46	9	5	3	0
18	Myricetin	1.39	318.24	8	6	1	1
19	Neoandrographolide	1.17	480.60	8	4	7	0
20	Pentagalloylglucose	2.76	940.68	26	15	16	3
21	Piperine	3.33	285.34	4	0	3	0
22	Quercetin	1.68	302.24	7	5	1	0
23	Rosmarinic acid	1.63	360.32	8	5	7	0
24	Rutin	-1.06	610.52	16	10	6	3
25	Torvoside	2.67	740.93	13	7	4	3

MW: molecular weight; RB: rotatable bond.

**Table 2:** AutoDock Vina docking results for antiviral phytochemicals against ZIKV NS3 helicase (PDB: 5JRZ).

S. no.	Phytochemicals	Binding affinity (kcal/mol)
1.	Ellagic acid	-9.9
2.	Hypericin	-9.8
3.	Pentagalloylglucose	-9.5
4.	Epigallocatechin gallate	-9.2
5.	Rutin	-9.1
6.	Glycyrrhizic acid	-9.1
7.	Mulberroside	-8.9
8.	Myricetin	-8.8
9.	Quercetin	-8.5
10.	Baicalein	-8.5
11.	Fisetin	-8.5
12.	Torvoside	-8.3
13.	Hyperoside	-8.1
14.	Kaempferol	-8.0
15.	Lupeol	-8.0
16.	Neoandrographolide	-8.0
17.	Rosmarinic acid	-8.0
18.	Apigenin	-7.9
19.	Chebulagic acid	-7.8
20.	Mimusopic acid	-7.7
21.	Berberine	-7.7
22.	Betulin	-7.6
23.	Curcumin	-7.5
24.	Geraniin	-7.4
25.	Piperine	-7.1

**Table 3:** Specific interaction type of phytochemicals with NS3 helicase of ZIKV (PDB: 5JRZ).

S. no.	Compounds	Interacting amino acids of NS3 helicase and distances	Hydrophobic contacts
1	Ellagic acid	Arg459 (2.1), Gln455 (2.4), Glu231 (1.8 & 1.9), Arg202 (2.1), Thr201 (2.5), Gly199 (2.8), Ala198 (2.8), Leu194 (2.0), and His195 (2.1)	Arg776, Thr790, Leu858, Cys775, Leu777, Lys745, Asp855, Thr854, Met766, Leu844, Val726, Met793, Cys797, Ala743, Leu792, Leu718, Met1002, and Gly796

2	Hypericin	Glu286 (2.7 & 3.4), Gly415 (2.6), Glu231, Thr201 (2.4), and Lys200 (2.2 & 2.3)	Gly415, Glu231, Ala416, Arg462, Arg202, Gly197, Asn417, and Gly199

3	Pentagalloylglucose	Asp410 (1.9 & 2.6), Met414 (2.0), Asp540 (1.9, 2.6 & 2.1), and Arg226 (2.0 & 2.1)	Asp291, Val227, Glu413, Arg388, Thr225, Ile411, Phe391, Cys262, Ala264, and Phe289

**Table 4:** Binding free energy calculation for ellagic acid, hypericin, and pentagalloylglucose through the MMPBSA method.

S. no.	Protein-ligand complex	Δ*E*_binding_ (kJ/mol)	Δ*E*_Electrostatic_ (kJ/mol)	Δ*E*_van der Waals_ (kJ/mol)	Δ*E*_polar solvation_ (kJ/mol)	SASA (kJ/mol)
1	Ellagic acid	−346.4 ± 7.1	−169.6 ± 7.2	−213.4 ± 9.6	69.2 ± 5.7	−32.6 ± 1.3
2	Hypericin	−343.7 ± 6.4	−167.5 ± 7.1	−211.3 ± 8.7	64.6 ± 4.3	−29.5 ± 2.3
3	Pentagalloylglucose	−338.5 ± 8.6	−164.9 ± 8.9	−209.7 ± 7.9	67.5 ± 8.1	−31.4 ± 7.6

**Table 5:** ADMET calculations.

Models	Ellagic acid	Hypericin	Pentagalloylglucose
BBB	BBB_positive_	BBB_negative_	BBB_positive_
HIA	HIA_positive_	HIA_positive_	HIA_negative_
Caco-2 permeability	Caco2_negative_	Caco2_positive_	Caco2_negative_
P-gp substrate	Substrate	Substrate	Substrate
P-gp inhibitor	Noninhibitor	Noninhibitor	Noninhibitor
Renal organic cation transporters (OCTs)	Noninhibitor	Noninhibitor	Noninhibitor
CYP450 2C9 substrate	Nonsubstrate	Nonsubstrate	Nonsubstrate
2D6 substrate	Nonsubstrate	Nonsubstrate	Nonsubstrate
3A4 substrate	Nonsubstrate	Nonsubstrate	Substrate
1A2 inhibitor	Noninhibitor	Inhibitor	Noninhibitor
2C9 inhibitor	Noninhibitor	Inhibitor	Noninhibitor
2D6 inhibitor	Noninhibitor	Noninhibitor	Noninhibitor
2C19 inhibitor	Noninhibitor	Inhibitor	Noninhibitor
3A4 inhibitor	Noninhibitor	Inhibitor	Noninhibitor
AMES test	Non-AMES toxic	Non-AMES toxic	Non-AMES toxic
Carcinogens	Noncarcinogens	Noncarcinogens	Noncarcinogens

## Data Availability

The data that support the findings of this study are available from the corresponding author upon reasonable request.

## Supplementary Materials

Supplementary Materials Figure S1: Plant-derived antiviral compounds: (a) apigenin, (b) baicalein, (c) berberine, (d) betulin, (e) chebulagic acid, (f) curcumin, (g) ellagic acid, (h) epigallocatechin gallate, (i) fisetin, (j) geraniin, (k) glycyrrhizic acid, (l) hypericin, (m) hyperoside, (n) kaempferol, (o) lupeol, (p) mimusopic acid, (q) mulberroside C, (r) myricetin, (s) neoandrographolide, (t) pentagalloylglucose, (u) piperine, (v) quercetin, (w) rosmarinic acid, (x) rutin, and (y) torvoside.
