# Supplementary material for: Ellagic Acid as a Potential Inhibitor against the Nonstructural Protein NS3 Helicase of Zika Virus: A Molecular Modelling Study
Source: Biomed Res Int. 2022 Aug 21;2022:2044577. doi: 10.1155/2022/2044577 (PMC9420600; doi:10.1155/2022/2044577)
Supplement: Supplementary Materials — Figure S1: Plant-derived antiviral compounds: (a) apigenin, (b) baicalein, (c) berberine, (d) betulin, (e) chebulagic acid, (f) curcumin, (g) ellagic acid, (h) epigallocatechin gallate, (i) fisetin, (j) geraniin, (k) glycyrrhizic acid, (l) hypericin, (m) hyperoside, (n) kaempferol, (o) lupeol, (p) mimusopic acid, (q) mulberroside C, (r) myricetin, (s) neoandrographolide, (t) pentagalloylglucose, (u) piperine, (v) quercetin, (w) rosmarinic acid, (x) rutin, and (y) torvoside. [file 2044577.f1.doc]

Ellagic acid as a potential inhibitor against the non-structural protein NS3 helicase of ZIKA Virus: A Molecular Modelling study

**Figure captions**

**Figure S1.** Plant derived antiviral compounds.


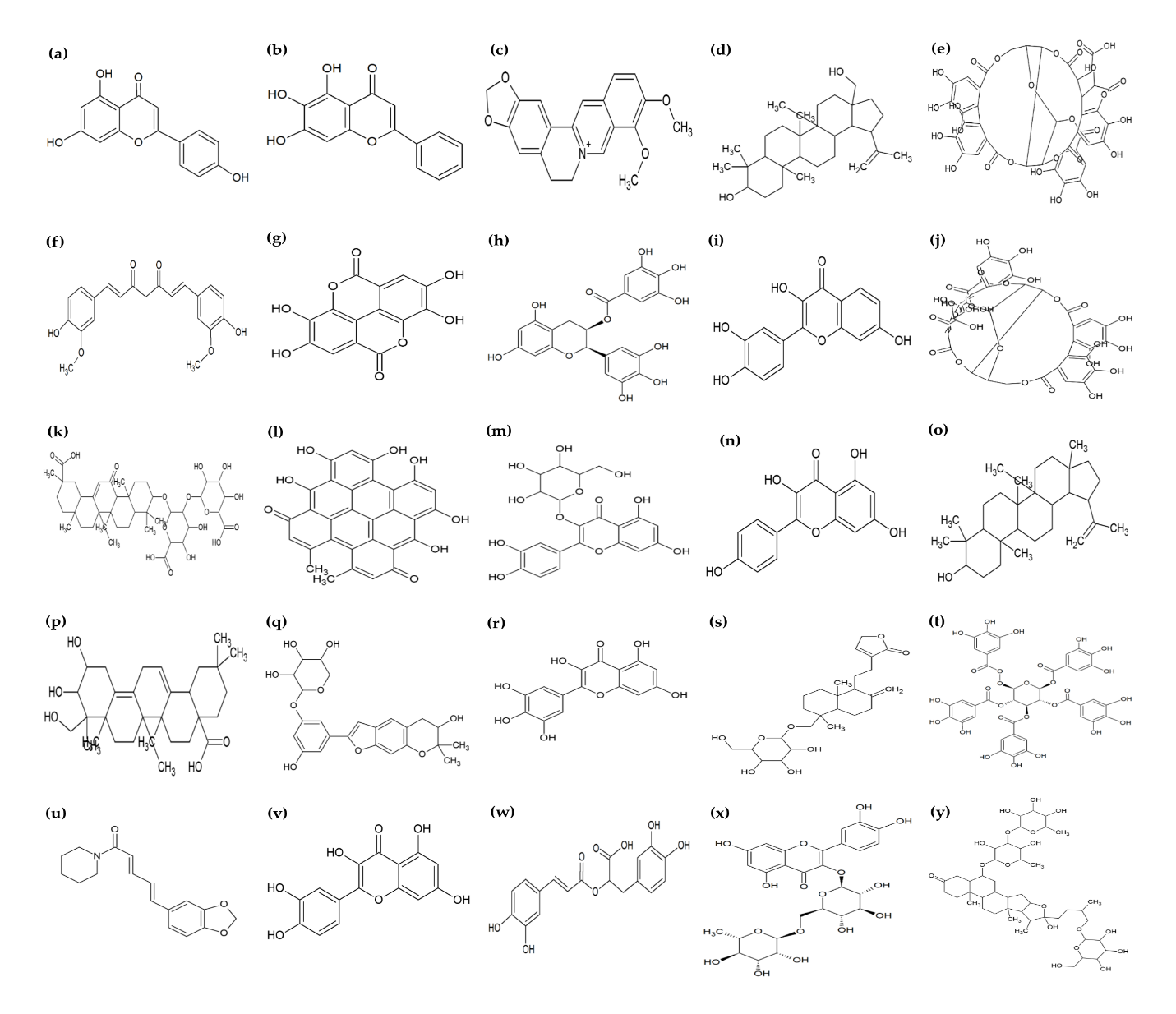


**Figure S1.** Plant derived antiviral compounds. (a) Apigenin (b) Baicalein (c) Berberine (d) Betulin

(e) Chebulagic_acid (f) Curcumin (g) Ellagic_acid (h) Epigallocatechin gallate (i) Fisetin (j) Geraniin (k) Glycyrrhizic Acid (l) Hypericin (m) Hyperoside (n) Kaempferol (o) Lupeol (p) Mimusopic acid (q) Mulberroside C (r) Myricetin (s) Neoandrographolide (t) Pentagalloylglucose (u) Piperine (v) Quercetin (w) Rosmarinic acid (x) Rutin (y) Torvoside
